# Sustainable nano-pesticide platform based on Pyrethrins II for prevention and control *Monochamus alternatus*

**DOI:** 10.1186/s12951-022-01409-6

**Published:** 2022-04-10

**Authors:** Yanxue Liu, Gehui Wang, Yixiao Qin, Long Chen, Chenggang Zhou, Luqin Qiao, Huixiang Liu, Chunyan Jia, Jiandu Lei, Yingchao Ji

**Affiliations:** 1 College of Plant Protection, Shandong Agricultural University, Tai’an, 271018 Shandong China; 2 Shandong Research Center for Forestry Harmful Biological Control Engineering and Technology, Shandong Agricultural University, Tai’an, 271018 Shandong China; 3 Taishan Scenery and Scenic Spot Area Management Committee, Tai’an, 271018 Shandong China; 4 Beijing Key Laboratory of Lignocellulosic Chemistry, College of Material Science and Technology, Beijing Forestry University, Beijing, 100083 China

**Keywords:** Stem-borer, Nano-pesticide, Enhanced bioactivity, Sustainable delivery, Bioassay

## Abstract

**Background:**

Pine wilt disease as a devastating forest disaster result from *Bursaphelenchus xylophilus* that spread by stem-borers *Monochamus alternatus* feeding on pine leaves, which has brought inestimable economic losses to the world's forestry due to lack of effective prevention and control measures. In this paper, we put forward a proposal for utilizing nanoHKUST-1 to encapusulate the Pyrethrins II that a nerve agent extracted from plant to control *M. alternatus*, including toxicity mechanism research, traceable biopesticide monitoring, and environment assessment for the first time. The highly biocompatible nanoHKUST-1 can solve the problems of poor water solubility, easy degradation and low control efficiency of Pyrethrins II.

**Results:**

The results illustrated the biopesticide loading efficiency of PthII@HKUST-1 reached 85% and the cumulative release of pH-dependent PthII@HKUST-1 was up to 15 days (90%), and also effectively avoid photodegradation (pH 7.0, retention 60.9%). 50 nm PthII@HKUST-1 made it easily penetrate the body wall of MA larvae and transmit to tissue cells through contact and diffusion. Moreover, PthII@HKUST-1 can effectively enhance the cytotoxicity and utilization of Pyrethrins II, which will provide valuable research value for the application of typical plant-derived nerve agents in the prevention and control of forestry pests. PthII@HKUST-1 as an environmentally friendly nano-pesticide can efficiently deliver Pyrethrins II to the larval intestines and absorbed by the larvae. PthII@HKUST-1 could also be transmitted to the epidemic wood and dead wood at a low concentration (10 mg/L).

**Conclusion:**

Here we speculate that nanoHKUST-1 will bring new opportunity to research biopesticide inhibition mechanism of different agricultural and forestry pests, which will break through the existing research limitations on development, utilization and traceable monitoring of biopesticide, especially for the study of targeting specific proteins.

**Graphical Abstract:**

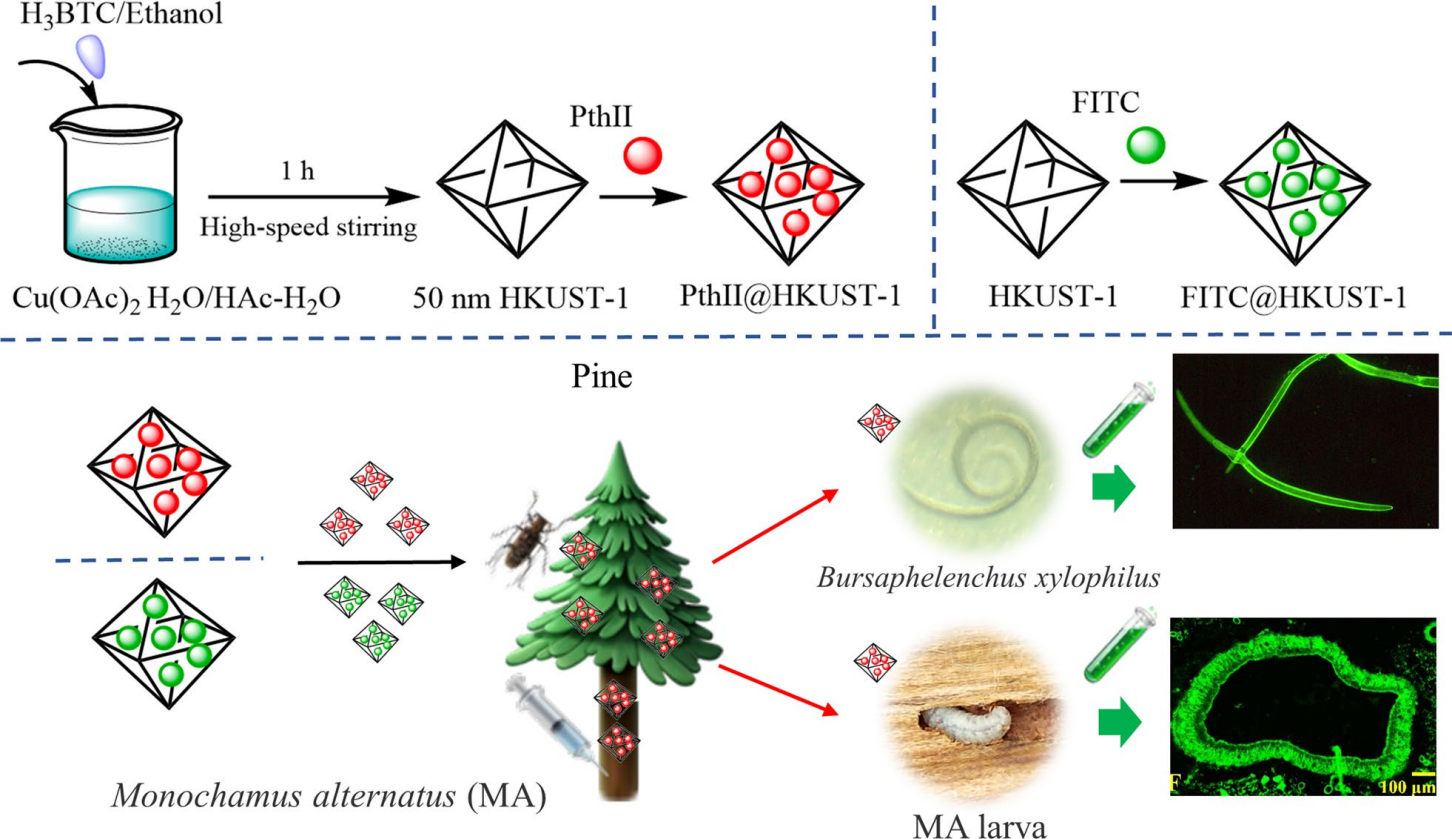

## Introduction

Stem-borers can seriously hinder the transportation of plant nutrients and water by drilling into branches and trunks, and harm a variety of economic and ecological tree species, leading to withering and even death [1, 2]. *Monochamus alternatus* Hope (MA) is not only an important stem-borer of pine, but also an important vector insect of pine wilt disease [3, 4]. Pine wilt disease, also known as pine cancer, can infect more than 50 kinds of pine trees, which has brought direct economic losses and ecological service value losses of more than 10 billion CNY per year in China [5–8]. In the process of pine wilt disease diffusion and infection, MA plays a key role in carrying, transmitting and assisting the pathogen *Bursaphelenchus xylophilus* to invade the host [5]. Therefore, effective prevention and control of MA is a key factor to cut off the transmission of pine wilt disease. At present, chemical control measures are considered to be one of the most effective and fastest solution to eliminate pests. Unfortunately, the current commercial insecticides cannot effectively control the occurrence and harm of *B. xylophilus* and MA because of low adhesion and transmission efficiency in the stem and branches of pine. The dosage of pesticides is multiplied to achieve the desired control effect, which not only increases the production cost but also causes the resistance of target organism to pesticides, and posing a serious impact on the safety of soil and groundwater [6–8]. Therefore, an efficient high drug loading drug delivery medium is considered to be urgently needed to be developed to effectively control Stem-borers MA.

In recently years, nanotechnology has gradually been applied in the agriculture and forestry pest control [9, 10]. Especially, nanotechnology shows excellent properties in the transportation of agrochemicals, including fertilizers [11], pesticides [12] and hormones [13]. In 2019, International Union of Pure and Applied Chemistry (IUPAC) ranked nano-pesticides as the top ten emerging chemical technologies that could change the world [14]. By use of special properties of nanomaterials, the defects of traditional formulations can be solved in a targeted manner [15]. For example, Zhao developed a hat-shaped carrier to prolong the retention time of the active ingredients on the leaf surface and release pesticides in a controlled manner by modifying nanomaterials [16]. Xu modified the thermosensitive material PNIPAm on the surface of PDA microspheres to prepare the pesticide temperature or near-infrared controlled release system [17]. Su et al. used a polymer biomaterial with excellent biocompatibility, bovine serum albumin, as pesticide Thiacloprid carrier to control trunk-boring pests [18].

**Fig. 1:**
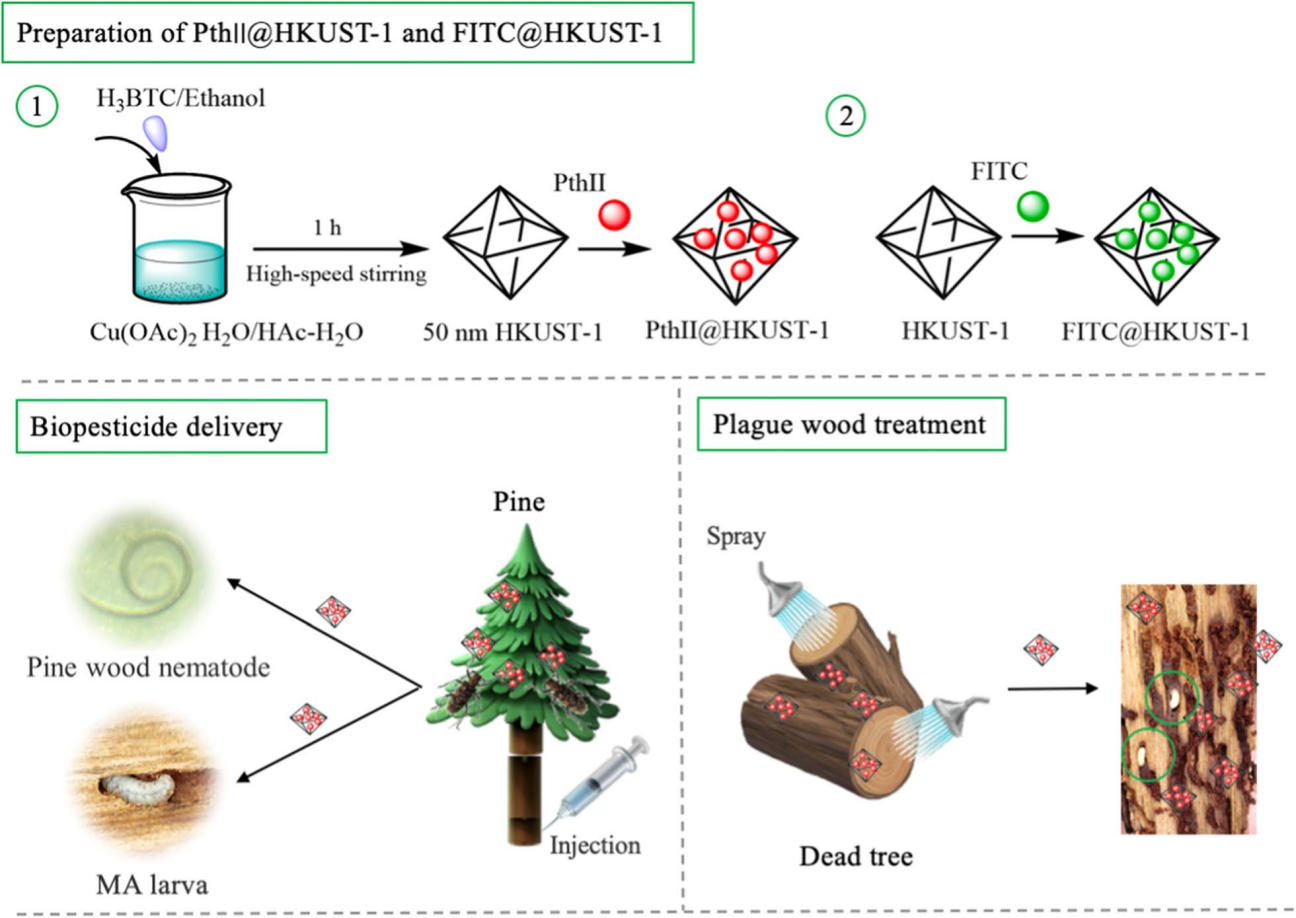
Schematic illustration of preparing PthII@HKUST-1 and FITC@HKUST-1, epidemic wood transmision of PthII@HKUST-1 that including traceable drug monitoring, and plague tree treatment

MOFs (Metal-organic frameworks) are a kind of organic–inorganic hybrid materials, also called coordination polymers, which are different from inorganic porous materials and general organic complexes [19, 20]. It has both the rigidity of inorganic materials and the flexibility of organic materials, and presents huge development potential and attractive development prospects in modern materials research because of open unsaturated metal sites. In addition, Pyrethrin, is an active ingredient with insecticidal effect isolated and extracted from *Pyrethrum cinerifoliun* [21]. It is internationally recognized as the safest and pollution-free natural insecticide to control agricultural and forestry crop pests [22]. HKUST-1 (Cu-MOFs) as a member of MOFs family has excellent catalytic and adsorption properties. Therefore, in this study, nanoHKUST-1 was utilized to encapusulate the Pyrethrins II (PthII) preparing a new PthII nano-pesticide to control MA. Meanwhile, the toxicity, transmission efficiency and environmental impact of PthII nano-pesticide were also evaluated in this study as is seen in Fig. 1.

## Materials and methods

### Materials and characterization

Pyrethrins II (PthII, 98%) was purchased from Shanghai Acmec Biochemical Co., Ltd (Shanghai, China); Trimesic acid (H_3_BTC, 98%), copper acetate (Cu(OAc)_2_⋅H_2_O, 98%) and acetic acid (HAc, > 99.8%) were provided by Shanghai Macklin Biochemical Co., Ltd. (Shanghai, China); Fluorescein isothiocyanate (FITC, > 95%) and trypan blue were supplied by Coolaber Science & Technology Co., Ltd (Beijing, China); DAPI Staining Solution and 4%Paraformaldehyde Fixative Solution were supplied by Shanghai Beyotime Bio-Technology Co., Ltd (Shanghai, China); DMEM medium and Fetal bovine serum (FBS) were obtained from ThermoFisher scientific Co., Ltd (Shanghai, China); Insect cell culture medium sf9 was purchased from Thermo Fisher Scientific (Shanghai, China). Nematode growth medium (NGM) was obtained by Shijiazhuang cmore Technology Co., Ltd (Shijiazhuang, China); CCK-8 kit was got from New Cell & Molecular Bio-Technology Co., Ltd; The *C. elegans*, the Bristol strain N2 and escherichia coli OP50 (*E. coli strain* OP50) were obtained from Sunybiotech Science & Technology Co., Ltd (Fujian, China); DF-1 cells were C/E chicken embryo fibroblasts which were resistant to endogenous ALV in the E subgroup, and were a gift from the Institute of Poultry Diseases and Tumors of the United States Department of Agriculture that was preserved by our laboratory for generations. Other reagents were from Beijing Aitemon Co., Ltd (Beijing, China).

X-ray diffraction (XRD) patterns were recorded with a Bruker D8 Discover 25 Advance diffractometer using nickel-filtered Cu Kα radiation (λ = 1.5406 Å). UV-visible spectra were recorded on a DaoJin UV-2550 spectrophotometer. Thermogravimetric analysis (TGA) was performed on a TG-DTA6300. Transmission electron microscopy (TEM) images was taken by a JEOL JEM 2100F at an accelerating voltage of 200 kV. Scanning electron microscopy (SEM) images were obtained by a JEOL JSM-6700F field-emission SEM with an accelerating voltage of 10 kV. Nitrogen sorption were performed in a MicroActive ASAP 2460 (2.02 version) adsorption apparatus at 77 K up to 1 bar to obtain pore volume and pore size by analyzing nitrogen adsorption and desorption isotherms. Confocal laser scanning microscope (CLSM) images were performed on a Leica TCS SP8 microscope. Fluorescent inverted microscope (Nikon TE2000), flow cytometer (BD LSRFortessa 4), stereo light microscope (SLM, Nikon, SMZ25), biological microscope (Yoke, XSP-8CA), high performance liquid chromatography (HPLC) is configured with Waters 1525 column oven and Waters 2998 UV detector. Cell viability was measured on a ThermoFisher Varioskan Lux Multifunctional microplate reader.

### Preparation of PthII@HKUST-1

HKUST-1 as a member of the MOFs is widely used in the fields of medicine, catalysis, membrane engineering, etc., which is chosen to load biopesticide into HKUST-1, hoping to avoid the application problems of small-molecule biopesticide on the basis of reducing environmental pollution, and realize the high-efficiency application of pesticides. However, the size of HKUST-1 is usually large, and although it is speculated that it can effectively kill the insect of MA, it may not be transmitted efficiently on the diseased pine trees. So, this will prompt us to consider this harsh problem. In this paper, nanoHKUST-1 was chosen for encapsulating PthII to research the toxicity mechanism, traceable biopesticide monitoring and environment assessment. Simply, 0.6 g Cu (OAc)_2_·H_2_O was dispersed in 10% aqueous acetic acid, and 0.42 g H_3_BTC/20 mL ethanol was added drop by drop under high speed stirring for 60 min. The blue precipitate was collected by centrifugation of 14,000 rpm for 30 min, and washed with ethanol several times, then the precipitation was replaced with deionized water twice, stored at  − 80 °C for 2 h before freeze-drying. The obtained lyophilized powder of nanoHKUST-1 was redispersed in 20 mg/mL PthII/ethanol and stirred for 48 h. The mixture was centrifuged to collect the precipitate and washed with ethanol several times to harvest PthII@HKUST-1.

### Preparation of FITC@HKUST-1

1 mg FITC was ultrasonically dissolved in 40 mL deionized water, and 50 mg nanoHKUST-1 powder was added to the FITC solution to stir at 300 rpm for 4 h in the dark. The product was centrifuged at 14,000 rpm for 20 min, and the supernatant was removed. The obtained precipitate was washed with deionized water several times to harvest FITC@HKUST-1.

### Detection of PthII@HKUST-1 biopesticide-loaded

5 mg nanoHKUST-1 powder was dispersed in different concentrations of PthII/ethanol solution, and stirred at 300 rpm for 48 h. After the reaction, the supernatant was collected at 14,000 rpm for 20 min to measure the content of free PthII using an UV spectrophotometer. The loading efficiency of PthII@HKUST-1 was calculated according to the following formula [18].

$$ {\text{Loading}}\,{\text{efficiency = }}\left( {\frac{{{\text{Weight}}\,{\text{of}}\,{\text{total}}\,{\text{PthII}}{ - }{\text{Weight}}\,{\text{of}}\,{\text{free}}\,{\text{PthII}}}}{{{\text{PthII@HKUST}}{ - }{1}}}} \right) \times 100\% $$

### Release performance of PthII@HKUST-1

The release curve of PthII@HKUST-1 was determined according to the method described in literature [23]. PthII@HKUST-1 powder was dispersed in PBS, transferred it to a dialysis bag after being ultrasonically dispersed, and put the dialysis bag into the deionized water. The release system was placed in a shaking box (150 rpm) for incubation. At different time points, the precipitate in the dialysis bag was collected by centrifugation, eluted with ethanol ultrasonic for three times, and centrifuged to collect the supernatant, and then the release amount of PthII in PthII@HKUST-1 was detected by high performance liquid chromatography (HPLC, Waters 2998 UV detector) after filtration with 0.22 µm filter membrane. The liquid chromatography detection conditions are as follows: mobile phases were acetonitrile (A) and 0.1% formic acid in water (B), gradient elution conditions: 0–10 min was 60–80% A, 10–10.1 min was 80–100% A, 10.1–15 min was 100% A,15–15.1 min was 100–60% A, 15.1–20 min was 60% A, with a flow rate 0.3 mL/min, injection volume 1 µL, column temperature 40 °C. The maximum absorption wavelength of PthII is 228 nm.

### Photodegradability of PthII@HKUST-1

1 mg PthII@HKUST-1 powder was ultrasonically dispersed in 10 mL of PBS with pH of 5.0, 7.0 and 9.0, and spread in a 9.0 cm petri dish, then natural dried in the dark. The petri dishes containing PthII@HKUST-1 were exposed to ultraviolet light for different time, Free PthII and PthII water emulsion (PthII/WE) were as a control. Free PthII was dissolved in ethanol and spread in 9.0 cm a petri dish, and PthII/WE was directly spread in 9.0 cm a petri dish. The anti-photolysis performance of PthII@HKUST-1 was measured by UV spectrophotometer at 228 nm, that is, the difference between the mass of PthII before irradiation and the remaining amount after irradiation.

### Biodistribution and cellular uptake

Firstly, ten of 5th instar MA larvae were placed in 6 cm petri dishes, respectively. The dispersed FITC@HKUST-1 (10 μg/mL) was sprayed on the larvae by small pressure spray can (soLo-408). After incubating for 5 min, the larvae are repeatedly rinsed with deionized water by small pressure spray can to remove non-adhered FITC@HKUST-1. The treated larvae were paralyzed with ethyl acetate and placed under a stereo light microscope (Nikon, SMZ25) to observe the fluorescence distribution on body wall and stomata. The main respiratory system of most insect larvae is through larval stomata, and a small amount spread through the body wall. Therefore, larval stomata and body wall gully area were focused on detection during the shooting. Secondly, the evenly dispersed FITC@HKUST-1 (10 μg/mL) solution was mixed with the artificial diet to feed MA larvae after starving for 24 h. After the larvae had fully fed for 1 h, the larvae were stored in 4% paraformaldehyde fixative solution for 48 h in the dark, and observed the fluorescence distribution of gut through dissecting intestines and making paraffin section. The fluorescence distribution of the larvae’s intestines was observed with a fluorescence microscope. Finally, 50 of 5th instar MA larvae were placed in 6 cm petri dishes, respectively. PthII@HKUST-1 (10 g/mL) was sprayed on the larvae by small pressure spray can and extend the crawling time of the larvae (1, 2, 3, 4 and 5 h) according to the previous operation. The epidermis of the larvae was dissected to observe the distribution of PthII@HKUST-1 on the epidermis through SEM.

In order to further study the cellular uptake of PthII@HKUST-1, DF-1 cells (C/E chicken embryo fibroblasts) were obtained as a model cell line, FITC@HKUST-1 was used to indirectly characterize the distribution of PthII@HKUST-1 on the surface of DF-1 cells. Firstly, DF-1 cells were inoculated into a 24-well plate at a concentration of 5 × 10^4^ cells/well, and cultured in DMEM medium containing 10% fetal bovine serum, 1% penicillin–streptomycin and 5% CO_2_ for 24 h at 37 °C. After incubating, the original medium of DF-1 cells were removed and added fresh medium containing FITC@HKUST-1 (10 μg/mL). After 4 h of incubation, DF-1 cells were washed with ice PBS for three times, and digest with 0.25% trypsin for 5 min, and then the reaction was terminated with ice PBS. DF-1 cells suspension was centrifuged at 2000 rpm for 5 min with the temperature of 4 °C to remove supernatant, and resuspend the cells in ice PBS. The cellular uptake of FITC@HKUST-1 was observed through CLSM.

### Cell morphology

Primary cell culture medium including 80% insect cell culture medium, 10% fetal bovine serum FBS and 10% phenylthiourea saturated solution, and subculture medium including 80% insect cell culture medium and 20% fetal bovine serum FBS, were ready for the following experiment. Under sterile conditions, the well-developed MA larvae were soaked in 70% ethanol for 20 min and absorbed the ethanol through the sterile absorbent paper. Then the insect needle was pierced into larval pronotum, and the insect blood cells were taken with a dot capillary. The blood cells were put into the primary cell culture medium and cultured under aseptic conditions at 27 °C for 7 days. After cultivation, the cell culture medium was transferred to the subculture medium and continued to incubate, and half of the subculture medium was taken out and replaced with fresh medium every week. PthII@HKUST-1, PthII/WE, Free-PthII and nanoHKUST-1 were added to the subculture medium with same concentration of PthII under aseptic conditions at 27 °C for cultivation. After 3 days of cultivation, the cell suspension was diluted with PBS and observed the cell morphology with a biological microscope.

### Cytotoxicity assessment of PthII@HKUST-1

DF-1 cells were inoculated into 96-well plates at a density of 5 × 10^3^ cells/well, and cultured in DMEM medium containing 10% fetal bovine serum, 1% penicillin–streptomycin and 5% CO_2_ for 24 h at 37 °C. After incubating, the original medium of DF-1 cells were removed and added fresh medium containing PthII@HKUST-1, and then PBS, PthII/WE and Free PthII were as control groups. Except for the PBS group, PthII/WE and PthII@HKUST-1 had the same amount of Free PthII. After 4 h of incubation, the medium of DF-1 cells were changed to the original medium to continue cultivate for 96 h. The medium was removed and added 20 µL, 5 mg/mL of CCK-8 to incubate for 4 h in the dark. The supernatant was removed and added 100 µL DMSO to detect the absorbance at 450 nm by a microplate reader. The calculation formula of cell viability is as follows:

$$ \left[ {\left( {{\text{A}}_{{{\text{Sample}}}} - {\text{A}}_{{{\text{Blank}}}} } \right)/\left( {{\text{A}}_{{{\text{Control}}}} - {\text{A}}_{{{\text{Blank}}}} } \right)} \right] \times 100\% $$

Among them, A_Sample_ is the absorbance value of the sample group, A_Blank_ is the absorbance value of the blank group, and A_Control_ is the absorbance value of the PBS group.

### Mechanism research of contact toxicity and stomachtoxicity

The contact toxicity and stomachtoxicity of PthII@HKUST-1 were determined by the biopesticide membrane contact method and the feed mixing biopesticide method [24, 25]. Firstly, the dispersed PthII@HKUST-1 solution was placed in a petri dish, and slowly spread the filter paper into it to make it completely soaked, then turned the filter paper sided down to the top, and placed the well-developed larva after starving for 24 h on the filter paper to observe the biopesticide intake of the larvae's abdomen. The well-developed 3rd-instar larvae were picked and placed on the filter paper, allowing them to crawl so that the larval epidermis completely contact PthII@HKUST-1 solution. Then the treated MA larvae were put in an artificial breeding box of 6 cm^3^. There were 30 larvae under each concentration gradient, and 10 larvae are a set of repeated group, and the control group was treated with water.

Feed mixing biopesticide method: 3 g of artificial feed were put into the breeding box, and 1 mL PthII@HKUST-1 solution was added. The well-developed 3rd-instar larvae were put into the breeding box. There were 30 larvae under each concentration gradient, and 10 larvae are a set of repeated group, and the control group was treated with water. The breeding boxes were placed in a constant temperature incubator with a temperature of 26 ± 1 ℃ and a relative humidity (RH) of 65 ± 10%. The survival rate of MA larvae was counted at 24 h and 48 h after feeding. If the larva did not move when touched with a brush. HKUST-1, PthII/WE and Free PthII were as control.

### Environmental assessment

Preparation of M9 buffer solution: Na_2_HPO_4_·12H_2_O (12.1 g), KH_2_PO_4_ (2.4 g), NaCl (4.0 g) and MgSO_4_·7H_2_O was dissolved in 1 L deionized water. Lysate solution: NaClO: 2 mol/L NaOH = 2:1 (v/v). The *Caenorhabditis elegans*, the Bristol strain N2 was cultured in inoculated *Escherichia coli* OP50 at 20 ℃. Adult nematodes in the egg-laying stage were washed with M9 buffer solution into the EP (Eppendorf) tube, then centrifuged to discard the supernatant, and M9 buffer solution was added to wash twice. The lysate solution was added and performed intermittent shaking fully lyse the nematodes. Then the M9 buffer solution was added and washed 3 times repeatedly to remove the lysate components. Finally, the M9 buffer solution was used to transfer the eggs that settle at the bottom of the centrifuge tube to an empty petri dish without medium, and incubated in an incubator at 20 °C for 12 h to obtain L1 instar larvae.

Before conducting the environmental safety assessment of nanomedicines, we firstly studied the stability of HKUST-1 in M9 buffer and also in nematode serum considering the stable delivery of HKUST-1 to nematodes, fetal bovine serum (FBS) was chosen for mimicing the in vivo serum environment of nematodes. Simply, HKUST-1 were dispersed in M9 buffer and 10% FBS for 7 days to observe their size changes by DLS detection. Then, HKUST-1, Free PthII and PthII@HKUST-1 were dispersed in M9 buffer solution, and mixed with *E. coli* OP50 solution at ratio 1:1 to obtain a culture solution with final concentrations of 0, 0.001, 0.01, 0.1, 1, 10, 100 and 1000 μg/mL. The prepared culture medium was spread evenly into the petri dish just poured with NGM medium and placed in a 37 °C incubator for 1 h. After the solution was dry, the petri dish was placed upside down and incubated for 11 h. The nematodes that reached L4 instar after being cultured for 2 days were transferred to a medium containing different concentrations of reagent. After 24 h of feeding, LC_50_, body length and width, and head swing frequency were measured.

Determination of LC_50_: different concentrations of reagent (200 μL) were added to the 96-well plate, and then 30 of L4 nematodes were picked into each well. Each concentration treatment was repeated three times. After contacting with the agent in the incubator at 20 °C for 24 h, counted dead nematodes under the microscope to calculate the LC_50_.

Determination of head swing frequency, body bending frequency and body length and width: the conventional application concentration 20 μg/mL of free PthII, HKUST-1 and PthII@HKUST-1 are configured to study the effect of three reagent on the body length, width and mobility of *C. elegans*. In one independent experiment, three L4 nematodes with the same activity were selected, one infected with Free PthII for 24 h, one infected with HKUST-1 for 24 h and one infected with PtII@HKUST-1 for 24 h, and then place them on three slides dripping with M9 buffer solution (60 μL). Each reagent was independently replicated 3 times, and total 90 nematodes were determined. After 1 min of stable adaptation, the head swing and body bending frequency of nematode were observed and recorded under a microscope within 1 min. The head of the nematode swings from one side of the body to the other, and the angle > 90° is defined as one head swing. A sine wave movement of the nematode relative to the long axis of the body is defined as a one body bending. In addition, the nematode to be tested was anesthetized, then observed and photographed with a microscope, and the body length and width was measured using Image J software.

### In-tree delivery of PthII@HKUST-1

Although it has been clarified that PthII@HKUST-1 could effectively kill MA larvae in the direct application process, it is still a big problem that cannot be ignored whether it can effectively penetrate the bark of the diseased pine into the trunk and spread through the water transport. After all, realizing the practical application of PthII@HKUST-1 is our core value of preparing nano-pesticides. As an important evaluation standard, transmission of PthII@HKUST-1 in tree trunks were studied in this paper. Experiment base on studying transmission of PthII@HKUST-1 was is located at Taishan Forest Area in the city of Tai'an, Shandong Province, China (117.06 °E, 36.23 °N). An electric drill was used to drill a hole with a diameter of 5 mm and a depth of 4 cm in the trunk at a 45-degree angle downward from the ground of 30 cm. First, FITC@HKUST-1 was used to inject trees and the diseased pine was sawn off after different processing time. The diseased pine was cut into small wooden pieces along the direction of the pine pith to observe the transmission of FITC@HKUST-1 through the small animal image. Second, PthII@HKUST-1 was injected with the concentration 1.0 mg/L, 10 mg/L and 50 mg/L, respectively, and PthII/WE with the same concentration as a control. After 3 days, sawdust was taken 5 cm above the punch at different depths. The method of subcritical water was used to extract PthII from wood chips. Simply, the sawdust was mixed with deionized water and put into a small polytetrafluoroethylene tank, then tanked into the reaction kettle and placed in a high-temperature furnace at 120 ℃ for 30 min. The reaction kettle was taken out, and quickly cooled, centrifuged to remove the aqueous solution. Sawdust was added methanol and mechanically shaken for 2 min, then centrifuged to retain the supernatant, continued to repeat the above operation 2 times. The methanol solution was collected to measure the content of PthII by an ultraviolet spectrophotometer. Third, the dead wood was sprayed with the concentration 1.0 mg/L, 10 mg/L and 50 mg/L, respectively, and PthII/WE with the same concentration as a control. After 3 days, sawdust was taken 5 cm above the punch at different depths. The method of subcritical water was also used to extract PthII from wood chips with the same operation method.

### Statistical analysis

All experiments in this work were repeated three times, and statistical analysis of the data was performed by analysis of variance (ANOVA), and all the data were subjected to normality and homogeneity tests using DPS v 7.05. All graphical data are reported as the mean ± standard deviation (SD). Significance levels were set at * *p* < 0.05.

## Results and discussion

### Characterization of PthII@HKUST-1

**Fig. 2:**
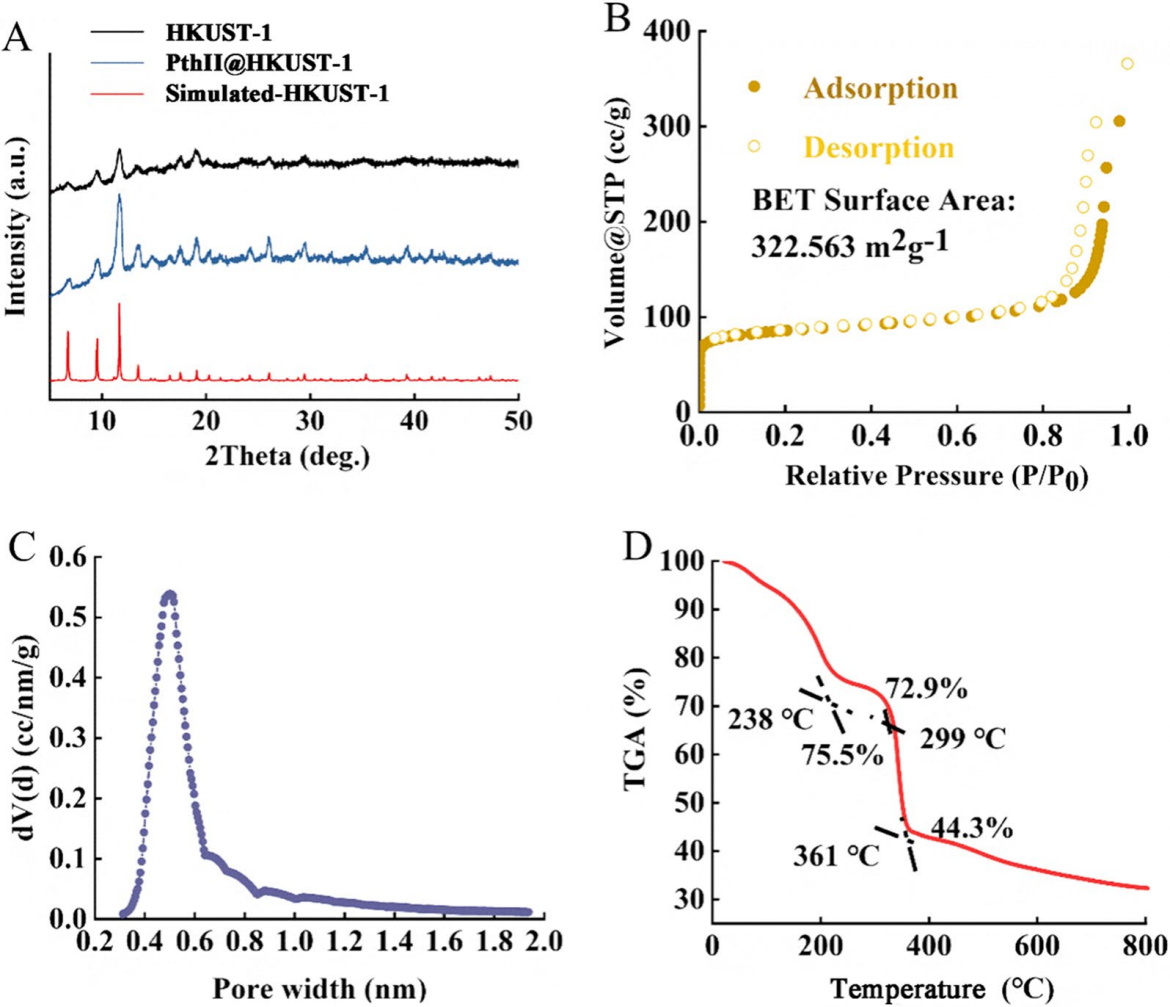
The XRD of simulated HKUST-1, HKUST-1 and PthII@HKUST-1 (**A**); BET (**B**), mesoporous distribution (**C**) and TGA (**D**) of HKUST-1

**Fig. 3:**
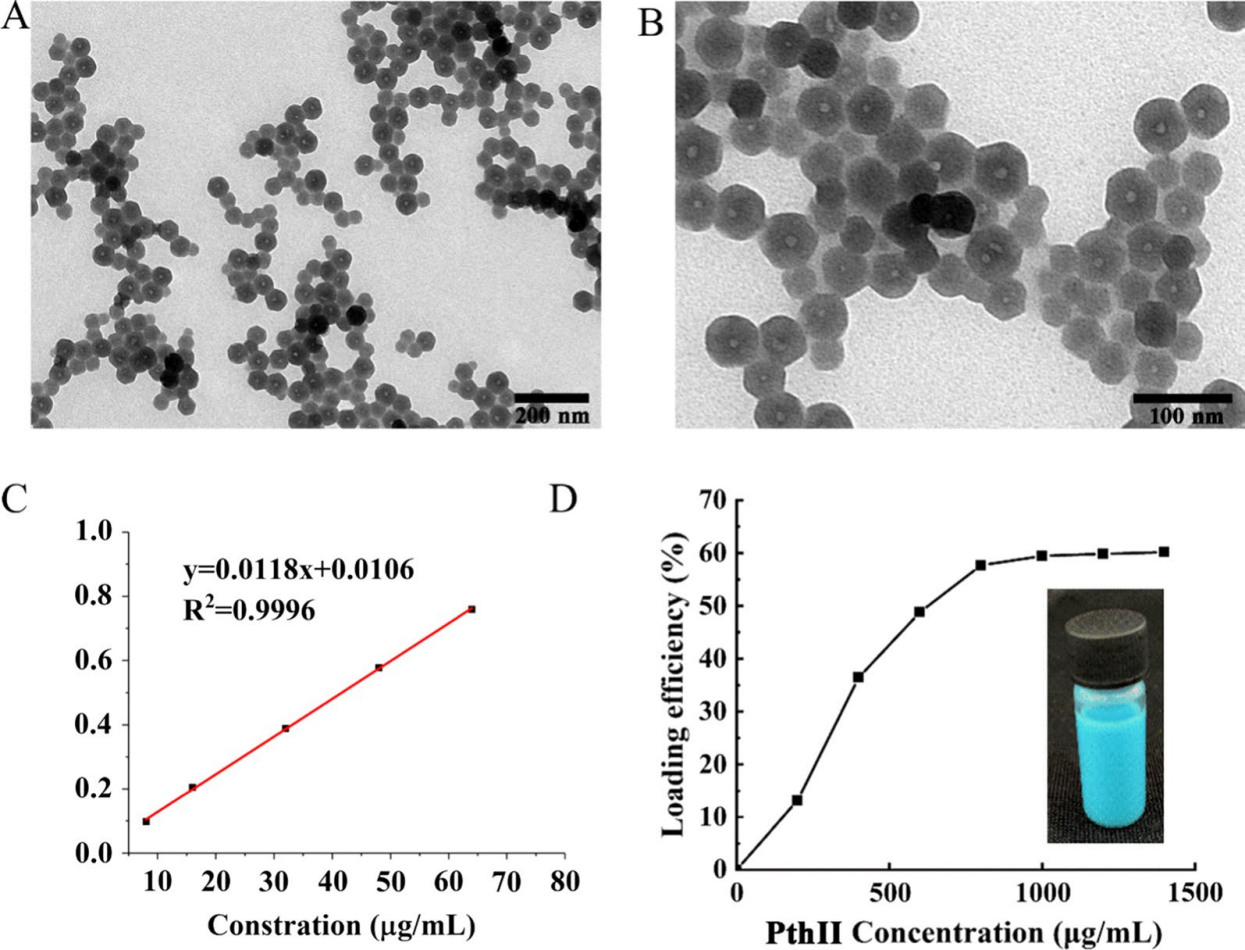
The SEM image (**A**) of PthII@HKUST-1, and magnified image (**B**); **C** The standard curve of PthII; **D** The loading efficiency of PthII@HKUST-1 and picture of PthII@HKUST-1

In this study, nanoHKUST-1 was synthesized by the method of coordination at room temperature, and PthII@HKUST-1 was prepared through adsorption to harvest blue powder. Figure 2A was the XRD of PthII@HKUST-1 and FITC@HKUST-1. It can be seen from the X-ray diffraction pattern that the peak positions of the characteristic peaks of PthII@HKUST-1 and FITC@HKUST-1 were basically consistent with those of the HKUST-1 curve, and the intensity of the PthII@HKUST-1 curve was similar to that of FITC@HKUST-1 curve, indicating that the purity and crystallinity of the sample obtained were close to HKUST-1, and Free PthII and FITC did not change the structure of HKUST-1 during the process of adsorption. From the Langmuir adsorption isotherm (Fig. 2B, nitrogen adsorption–desorption) and pore size distribution (Fig. 2C) of HKUST-1 that HKUST-1 was a microporous structure with a specific surface area of 322.5 m^2^/g. HKUST-1 has excellent adsorption properties because of it’s open unsaturated copper metal sites, which brings opportunities for the successful adsorption of PthII. The changes in thermal weight loss of PthII@HKUST-1 with the temperature range of 0–800 ℃ were observed through the thermal weight loss curve (Fig. 2D). The quality of PthII@HKUST-1 reduced by 24.5% in the heating range of 0–238 °C, which may be due to the decomposition of PthII (thermally labile compounds). The TGA curve of PthII@HKUST-1 was relatively flat during the heating range of 238–299 °C, and dropped significantly in the heating range of 299–361 °C. The quality of PthII@HKUST-1 lost heavily which may be caused by the collapse of the internal structure of the sample from 299 °C. SEM image of PthII@HKUST-1 was shown in Fig. 3A, B, the dynamic diameter of PthII@HKUST-1was about 50 nm and uniform particle size, laying the foundation for the next assessment of biopesticide-loaded.

### Detection of PthII@HKUST-1 biopesticide-loaded

The calibration curve of PthII was linear in the concentration range of 8–80 μg/mL, the regression equation was y = 0.0118x + 0.0106, and the correlation coefficient was 0.9996 (Fig. 3C). Generally speaking, the biopesticide loading capacity directly affects the field application dose of pesticides. The larger the biopesticide loading efficiency, the easier it is to meet the needs in field [26]. NanoHKUST-1 powder was dispersed in different concentrations of PthII ethanol solutions and drawn pictures according to the relationship between loading efficiency and PthII concentration. The loading efficiency gradually increased with the enhanced concentrations of PthII, and the loading efficiency reached 85% when the concentrations of PthII were 5 mg/mL (Fig. 3D). However, the biopesticide loading rate unchanged after continuing to increase the concentration of PthII, suggesting that nanoHKUST-1 has reached exhaustion adsorption.

### Release performance of PthII@HKUST-1

**Fig. 4:**
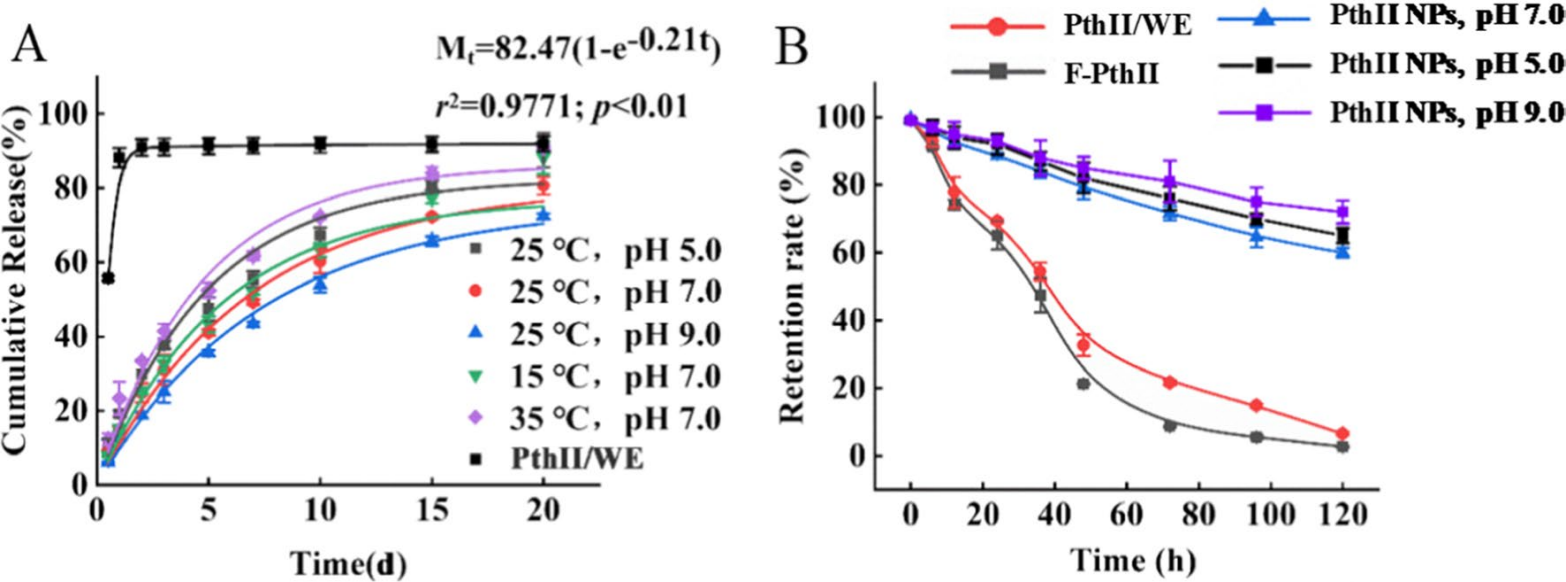
**A** The release curve of PthII@HKUST-1 with 15 °C, 25 °C and 35 °C under pH of 5.0, 7.0 and 9.0, and PthII@WE as a control; **B** The retention rate of Free PthII, PthII/WE and PthII@HKUST-1 with pH of 5.0, 7.0 and 9.0 after being exposed to ultraviolet light

The difference in release performance of biopesticide can greatly affect the control effect of pesticides in the field. Extending the slow release of pesticides can reduce the frequency of pesticide application, and reduce large-scale environmental pollution caused by one-time release, and then achieve long-term effective prevention and control of target organisms [27]. This study simulated the environmental temperatures and pH values common in agriculture and forestry to explore the release performance of PthII@HKUST-1. First, the release performance of PthII@HKUST-1 at different temperatures was explored (Fig. 4A), the release of PthII@HKUST-1 belonged to the first-order release equation, and the fitting equation was Mt = 82.47(1-e-0.21t), the cumulative release exceeded 15 d, and the total cumulative release exceeded 90%. In contrast, PthII/WE treatment group suggest no sustained release properties, and the total release reached 88.4% in just one day. Compared with the current traditional PthII/WE, PthII@HKUST-1 had a more stable release and could be used to continuously control pests. In addition, it could be seen that under different temperature conditions, the release rate had changed. The higher the temperature, the faster the release rate. The release rate at 35 °C was faster than 25 °C and 15 °C, but the total cumulative release had no significant change, indicating that the release process of temperature in PthII@HKUST-1 must be considered. Otherwise, the cumulative release of pH 5.0 (91.9%) was higher than pH 7.0 (83.5%) and pH 9.0 (75.3%), suggesting that the acid and alkali condition of the soil was also a crucial factor for the controlled release of PthII@HKUST-1. At the same time, as a natural extract, PthII are unstable to acid, alkali, and light, which is the main reason why PthII is so noticeable and powerless. While nanoHKUST-1 effectively protects PthII from environmental interference. Whether the release is in line with expectations is an important indicator of the formulation process screening (Fig. 4B). Usually, multiple applications are often required to achieve the desired control effect due to the short release time of current commercial formulations, which leads to waste of active ingredients and increased costs. Under different temperature and pH conditions, PthII@HKUST-1 had a stable release process with a long release period and a high cumulative total release. Therefore, it is considered that PthII@HKUST-1 have a good release performance under common natural environments to achieve continuous prevention and control of harmful organisms. Therefore, the development of PthII@HKUST-1 is of great significance for reducing the pressure of agriculture on the environment and reducing the amount of traditional pesticides [28].

### Photodegradability of PthII@HKUST-1

It is necessary to consider the anti-photolysis effect of nanoHKUST-1 on the Pth II when preparing PthII@HKUST-1. The retention curve of PthII exposed to ultraviolet light clarified the protective effect of PthII@HKUST-1 on PthII (Fig. 4B). Obviously, the retention rate of PthII gradually decreased with the extension of the treatment time, and PthII@HKUST-1 (pH 9.0) > PthII@HKUST-1 (pH 5.0) > PthII@HKUST-1 (pH 7.0) > PthII/WE > Free PthII. The photodegradability of Free PthII (retention 2.59%) and PthII/WE (retention 6.51%) was far greater than that of PthII@HKUST-1 (pH 7.0, retention 60.9%), meaning that the nanoHKUST-1 had a positive effect on the anti-photolysis of PthII. The retention rate of PthII in the pH 7.0 and pH 9.0 was higher than that in the pH 5.0, implying that photodegradability of PthII@HKUST-1 depended on pH. The weak degradation of nanoHKUST-1 under acidic conditions promoted the release of PthII with the prolongation of the treatment time, and increased the photolysis rate, and then reduced the retention rate of PthII. The excellent anti-photolysis performance of PthII@HKUST-1 was mainly attributed to its porous structure, and PthII was absorbed on the pores inside nanoHKUST-1 during the preparation of PthII@HKUST-1, which naturally shielded the exposure of ultraviolet light.

### Biodistribution and cellular uptake

**Fig. 5:**
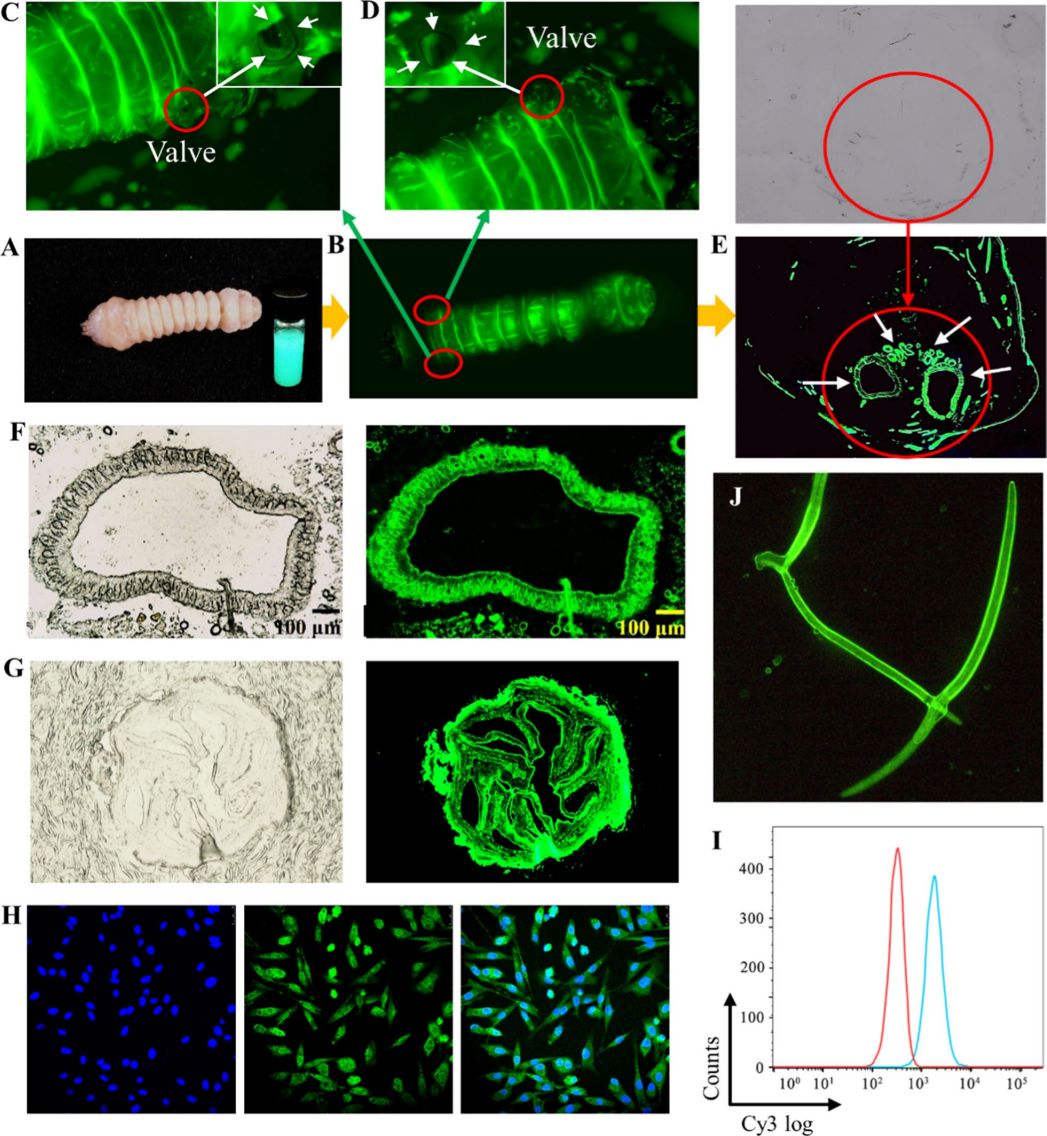
**A** Picture of FITC@HKUST-1 and MA larva after treatment with FITC@HKUST-1; **B** Body wall fluorescence distribution of MA larvae by SLM, and magnified image of larval stigma on the left (**C**) and right sides (**D**); **E** Fluorescence distribution of the middle cross section of MA larva; **F** Bright field image and fluorescence distribution image of the midgut cross section; **G** Bright field image and fluorescence distribution image of the cecum cross section; **H** The cellular uptake of FITC@HKUST-1 to DF-1 cells (green), DAPI stains the nucleus (blue), and merge image; **I** The uptake rate of FITC@HKUST-1 was detected by flow cytometry; **J** Fluorescence distribution of pine wood nematode after treatment with FITC@HKUST-1

In order to further verify the biodistribution of PthII@HKUST-1 to MA larvae, FITC-labeled nanoHKUST-1 were used for fluorescence test. Fluorescent microscope and SEM were used to explore the coverage, permeability and adhesion of PthII@HKUST-1 on the larval epidermis. Figure 5A was the MA larvae that treated with FITC@HKUST-1. Under the dark field excitation light of 488 nm, it was clearly observed that the morphology of MA larvae was successfully labeled with fluorescence (Fig. 5B), and there was fluorescence distributed on the entire epidermal structure of the larvae, especially in the folds, where the fluorescence was intensified, meaning that nanoHKUST-1 could be transferred to subcutaneous tissue through the body wall from the epidermis to the and be enriched in the body. After crawling for a period of time, the fluorescence did not fall off, indicating that FITC@HKUST-1 was of good adhesion on the larval epidermis. There were a large number of fluorescent particles distributed around the valve on both sides of the larva. As we all know, the respiration of insect larvae mainly depends on the larval stigma, followed by the body wall, so we enlarged the larval stigma of the MA larva to observe whether there is fluorescence distribution (Fig. 5C, D). As we expected, the larval stigma of the MA larva was enriched with fluorescence and spread to the depths when MA larva breathed followed by the valve stigma opened and close. We speculated that these larval stigmas would be the main channel for FITC@HKUST-1 to enter the body of larvae. It also confirmed that FITC@HKUST-1 was of good permeability and passed through the epidermal structure in a very short time, implying that PthII@HKUST-1 could effectively achieve contact toxicity. This speculation will be verified in the toxicity mechanism of PthII@HKUST-1. In addition, through observing the cross-sectional fluorescence distribution of the MA larva, the intestinal and dorsal blood vessels inside were clearly observed from Fig. 5E, revealing the fact that FITC@HKUST-1 was delivered from the body wall to the body tissue. The dissected intestine also fully confirmed this point (Fig. 5F). However, can MA larvae that feed with FITC@HKUST-1 reach to the intestine smoothly without being quickly digested by the stomach? The intestine of MA larvae was observed after dissection through a fluorescence microscope, and it was noticed that the intestine showed an enhanced distribution of green fluorescence, which indicated that biopesticide delivery from the stomach to the intestine could be achieved by feeding. In order to further observe the distribution of fluorescence inside the intestine, the cross-section of the intestine was sliced. From Fig. 5G, there was obvious fluorescence inside the cecum of MA larvae, and the outline was clearly visible, which provided a basis for the delivery of PthII.

Why can FITC@HKUST-1 play the role of contact toxicity and stomachtoxicity to MA larvae at the same time? Why can FITC@HKUST-1 continue to maintain the fluorescence intensity or efficacy? Why is FITC@HKUST-1 not digested in the stomach? This series of questions may come to the readers clearly. The answer to these questions may be explained why nanoHKUST-1 can efficiently realize the biological distribution in the MA larvae. From the previous release research, nanoHKUST-1 has sustained release performance under weak acid and weak alkali. Its sustained release is based on the degradation of nanoHKUST-1, which can answer the second and third questions. Moreover, because of the particle size advantage of nanoHKUST-1 (50 nm), makes it easily penetrate the body wall of MA larvae and transmit to tissue cells. This phenomenon can be further proved from Fig. 5H. We used avian DF-1 cells as model cells and observed the uptake of FITC@HKUST-1 on cells through CLSM. FITC@HKUST-1 was fully adsorbed to the cell surface and exhibited enhanced fluorescence uptake. The uptake rate of cells detected by flow cytometry was as high as 90–100% (Fig. 5I). It was obvious that nanoHKUST-1 extremely promoted the delivery and sustained release of PthII in MA larvae. Furthermore, FITC@HKUST-1 treated BX also observed satisfactory fluorescence distribution (Fig. 5J), BX was adhered a large amount of FITC@HKUST-1, indicating that nanoHKUST-1 can penetrate BX epidermal cells into the body.

**Fig. 6:**
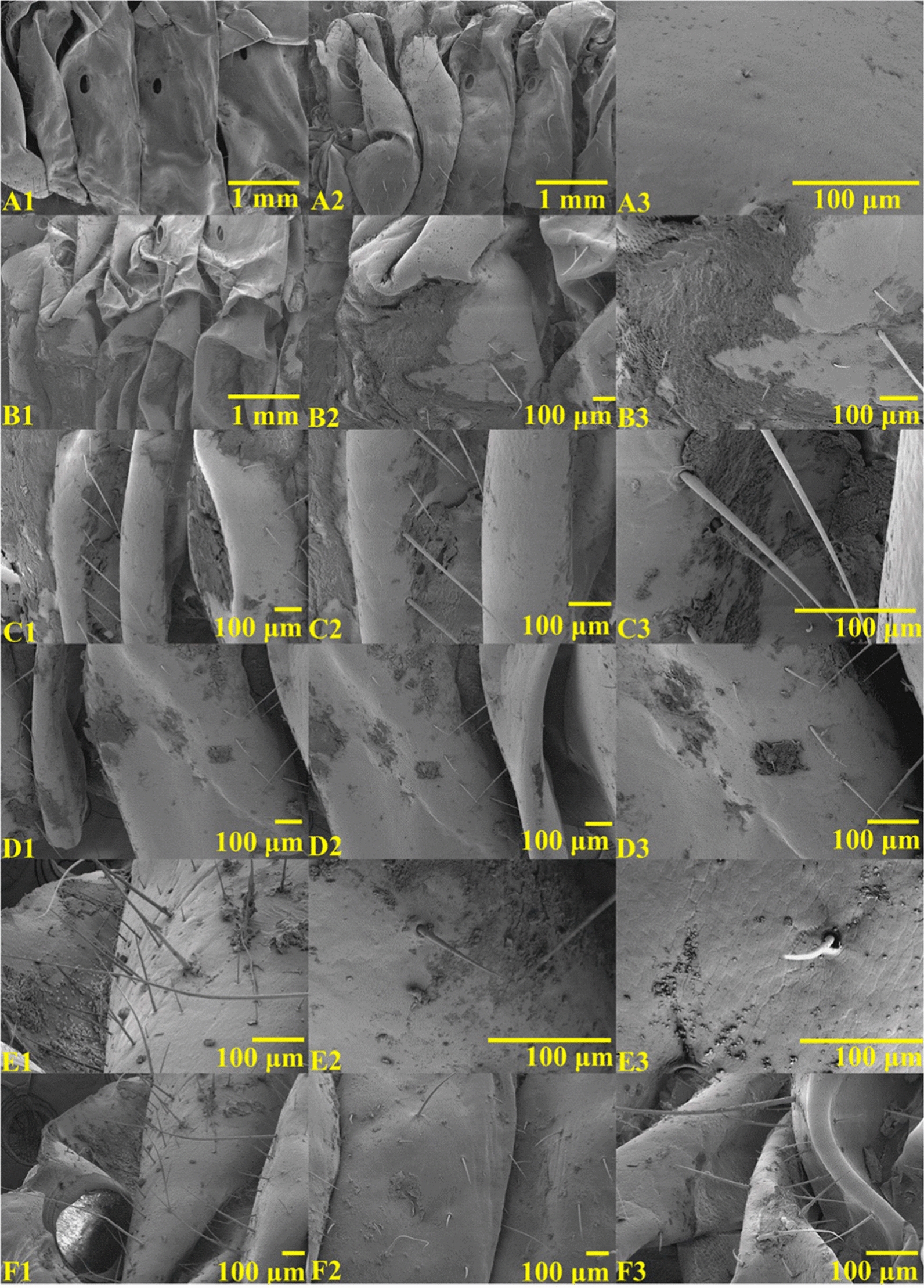
The SEM images of larval epidermis (**A1–A3**), the SEM images of larval epidermis treated by PthII@HKUST-1 for 1 h (**B1–B3**), 2 h (**C1–C3**), 3 h (**D1–D3**), 4 h (**E1–E3**), and 5 h (**F1–F3**)

Furthermore, the process of PthII@HKUST-1 passing through the epidermis into the larval body was observed by SEM (Fig. 6). The untreated larval epidermis structure was wrinkled and coarse after magnificatio, which brought conditions for the attachment of PthII@HKUST-1 (Fig. 6A1–A3). PthII@HKUST-1 was adhered to the larval epidermis after treatment (Fig. 6B1–B3), which not only covered a wide range, but also did not fall off after moving. PthII@HKUST-1 could be well embedded in the texture structure on the larval epidermis and were not easy to fall off. As the treatment time increased, it could be clearly observed that PthII@HKUST-1 on the larval epidermis gradually decreased (Fig. 6C1–D3), which mainly due to the ability of penetrated the epidermal structure and entered the larval body. A little PthII@HKUST-1 could be observed to adhere to the larval epidermis, which indicated that PthII@HKUST-1 might pass through the larval epidermal structure and got into the larval body (Fig. 6E1–F3).

### Cell morphology

**Fig. 7:**
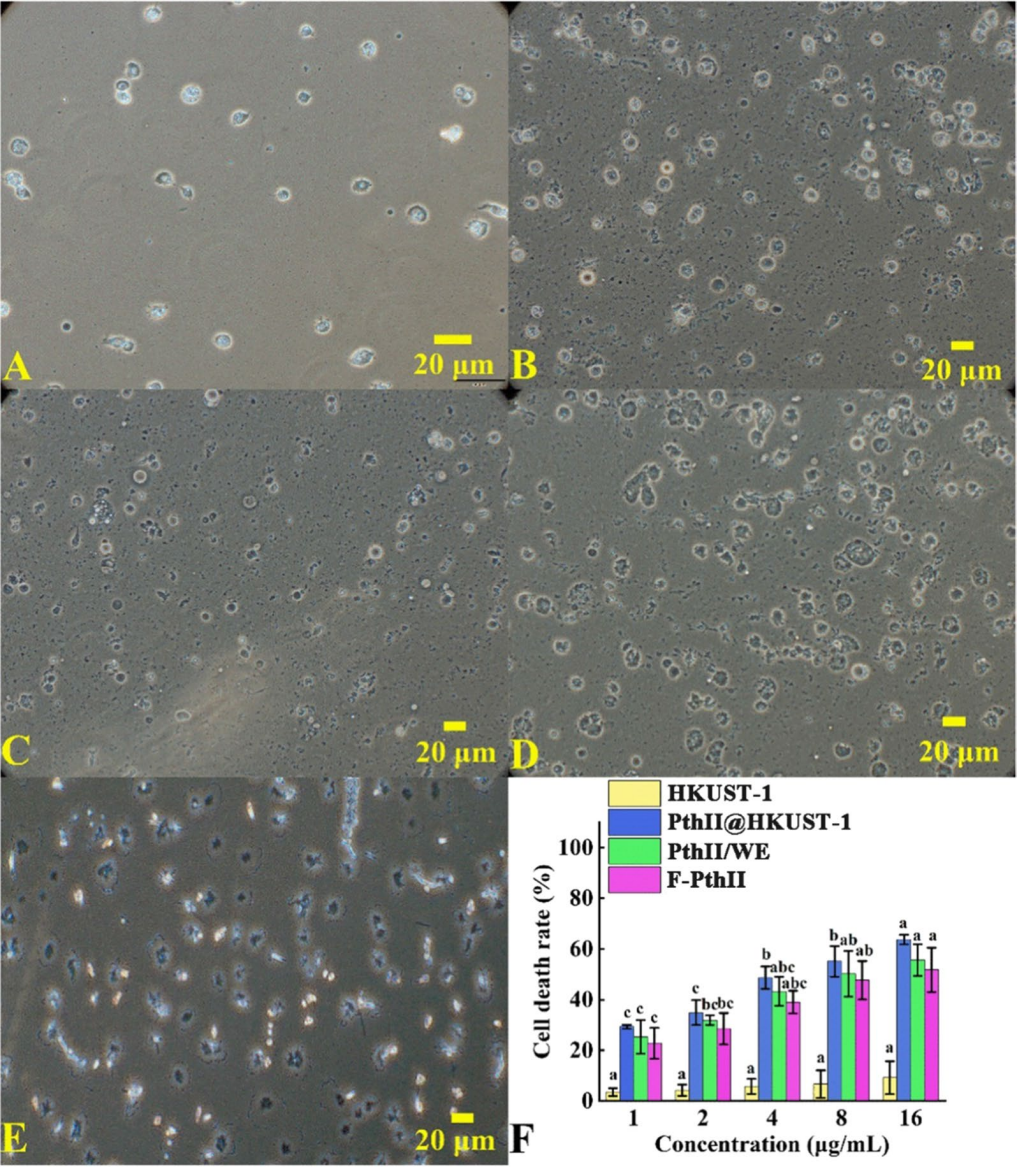
The normal blood cell (**A**) of MA larva, the cellular morphology after treated by HKUST-1 (**B**), Free PthII (**C**), PthII/WE (**D**), and PthII@HKUST-1 (**E**); **F** The cell death rate in different concentrations of HKUST-1, Free PthII, PthII/WE, and PthII@HKUST-1, data in the figure are mean ± SD of three replications (n = 3), different letters indicate significant differences (ANOVA, *p* < 0.05)

Due to effective cell uptake of PthII@HKUST-1, it could increase the cytotoxicity and exert the bioactivity [29, 30]. The effects of PthII@HKUST-1, PthII/WE, Free PthII and HKUST-1 on cell morphology were explored. As can be seen in Fig. 7A, normal blood cells of insects were nearly round rich in cytoplasm and the cell structure was intact without rupture. The larval blood cells treated with HKUST-1 had no obvious changes in morphology and still maintained a complete cell structure (Fig. 7B). After treated with PthII/WE and Free PthII, most parts of cells morphology had not changed, and only a small number of cells ruptured (Fig. 7C, D). However, after treated with PthII@HKUST-1, the cell morphology significantly changed (Fig. 7E). There were almost no complete blood cells, and the cells seriously deformed and ruptured. With the increasing cell uptake of PthII@HKUST-1, the cytotoxicity of PthII also enhanced, thereby causing significant changes in cell morphology.

### Cytotoxicity assessment of PthII@HKUST-1

In order to verified the above conclusions, the cytotoxicity of PthII@HKUST-1, PthII/WE, Free PthII and HKUST-1 was measured the in vitro (Fig. 7F). With the increasing of the concentration, the cytotoxicity of HKUST-1 was low and less than 10%. Compared with the PthII/WE and Free PthII, the cell cytotoxicity of PthII@HKUST-1 was the highest at the same concentration. When the concentration was 1 μg/mL, the apoptosis rate of PthII@HKUST-1, PthII/WE and Free PthII were 17.07%, 11.05% and 8.56%, respectively. The apoptosis rate of the PthII@HKUST-1 treatment group was 1.9 times that of the Free PthII. When the concentration of 16 μg/mL, the apoptosis rate of PthII@HKUST-1, PthII/WE and Free PthII were 97.37%, 87.17%, and 79.13%, respectively, and the gap was significantly reduced. In summary, PthII@HKUST-1 can effectively enhance the cytotoxicity and utilization of PthII, which will provide valuable research value for the application of typical plant-derived nerve agents in the prevention and control of forestry pests.

### Mechanism research of contact toxicity and stomachtoxicity

**Fig. 8:**
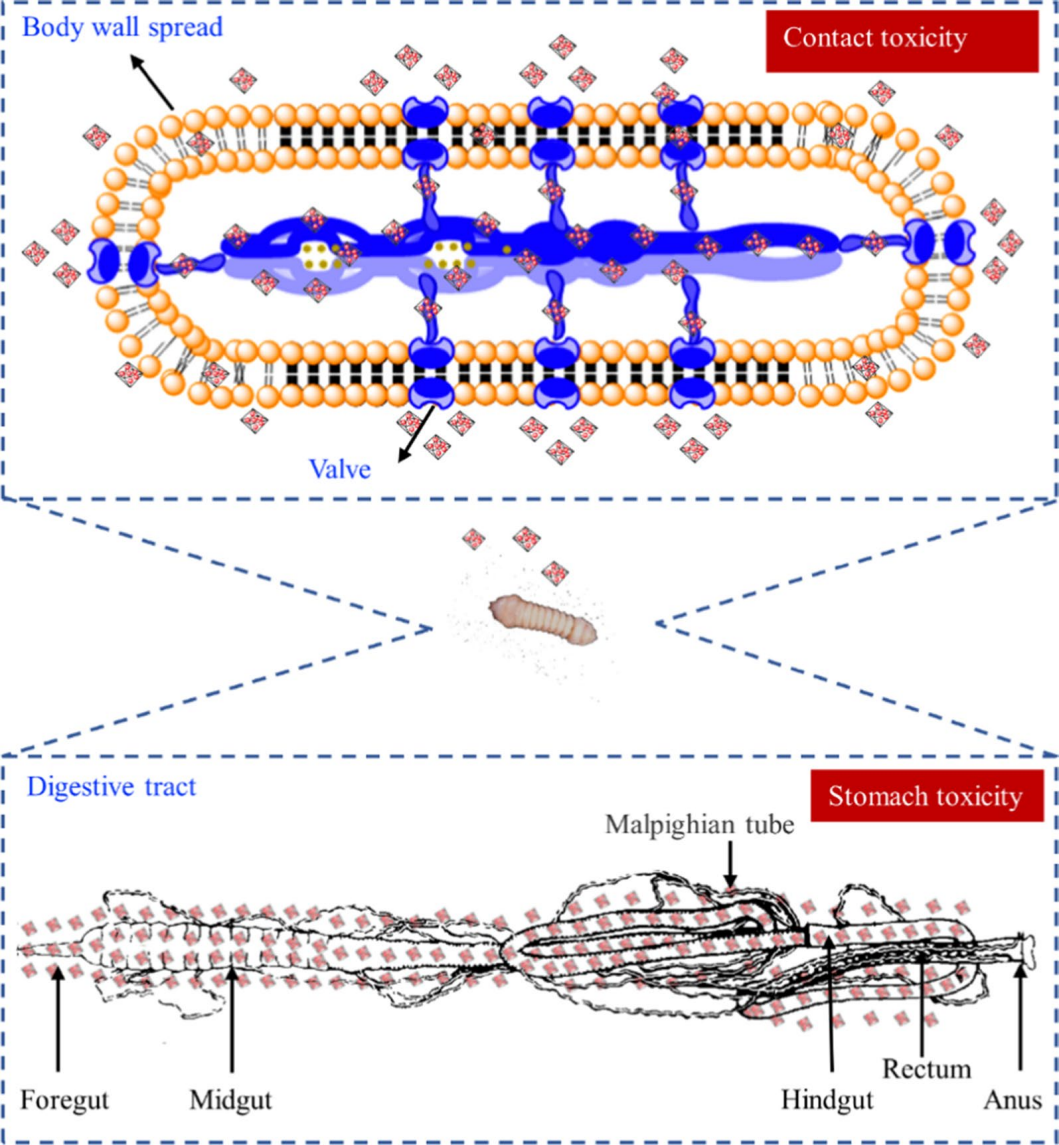
Toxicity mechanism studies of PthII@HKUST-1. **A** Contact toxicity and **B** stomach toxicity

**Fig. 9:**
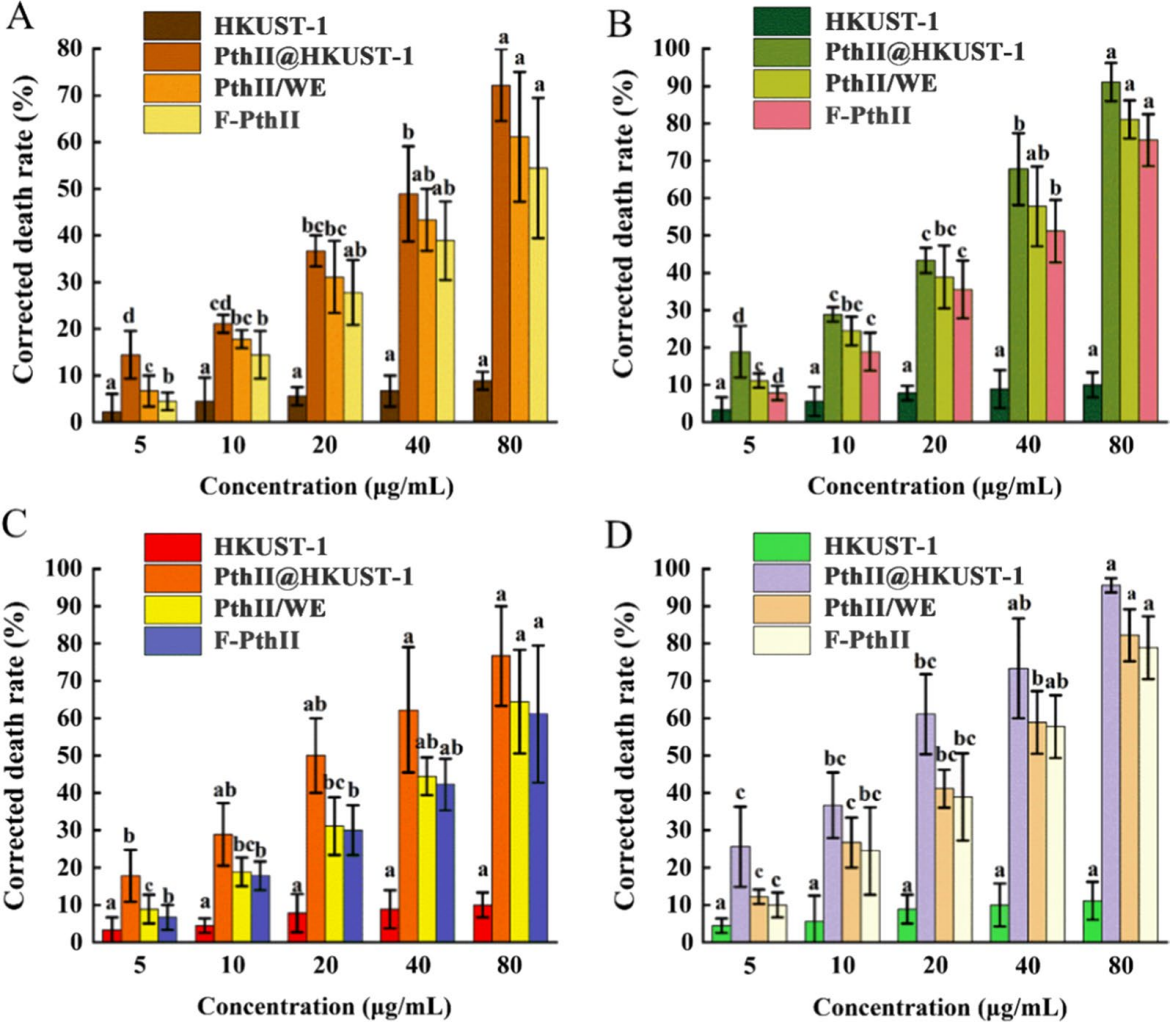
The contact toxicity in different concentrations of PthII@HKUST-1 at 24 h (**A**), 48 h (**B**), and the stomach toxicity at 24 h (**C**), 48 h (**D**). Data in the figure are mean ± SD of three replications (n = 3), different letters indicate significant differences (ANOVA, *p* < 0.05)

Contact toxicity refers to the pesticide entering the pest body by contacting the epidermis of the insects to exert its activity [31]. Due to the poor dispersion of traditional formulations, and the unsatisfactory coverage, permeability and adhesion, the active ingredients could not reach the site of action through the larval epidermis, resulting in unsatisfactory contact toxicity [32]. Stomachtoxicity refers to that the pesticide enters the body through the mouthparts of pest and exerts its activity during the feeding process [31]. However, the limited life of active ingredients, which results in the failure to reach the larval intestine efficiently and to be absorbed by pest [33]. Mechanism research of contact toxicity and stomachtoxicity of PthII@HKUST-1 was shown in scheme Fig. 8. The enhancement of PthII@HKUST-1 for contact toxicity and stomach toxicity was researched at the individual level, the data of which was listed in Fig. 9. The corrected mortality of HKUST-1 was the lowest among the four treatment groups whether the mode of action was contact toxicity or stomach toxicity. At the highest concentration, the corrected mortality was about 9.1%, indicating that the carrier has a low lethality rate to larvae. After 24 and 48 h treatment under the five concentrations, the contact toxicity of PthII@HKUST-1 had the highest lethality rate, followed by PthII/WE and PthII (Fig. 9A, B). The lethality rate of the PthII@HKUST-1 treatment group was increased by 2.4 times after treating with 5 μg/mL, while the contact toxicity was increased by 1.2 times after treating with 80 μg/mL, which was consistent with the in vitro cytotoxicity. The stomachtoxicity was consistent with results of the contact toxicity (Fig. 9C, D). The PthII@HKUST-1 had the highest corrected mortality of 25.6%, 36.7%, 58.9%, 73.3%, and 95.6%, respectively, and the average toxicity increased by 1.62 times. The lethality rate of the stomachtoxicity was higher than contact toxicity. In general, the prepared PthII@HKUST-1can effectively improve the contact toxicity and stomachtoxicity of PthII. PthII@HKUST-1 has a good dispersibility and efficiently delivers PthII to the larval intestines and absorbed by the larvae. After entering the larvae, PthII@HKUST-1could effectively increase the cell uptake rate, thereby increasing cytotoxicity.

### Environment assessment

**Fig. 10:**
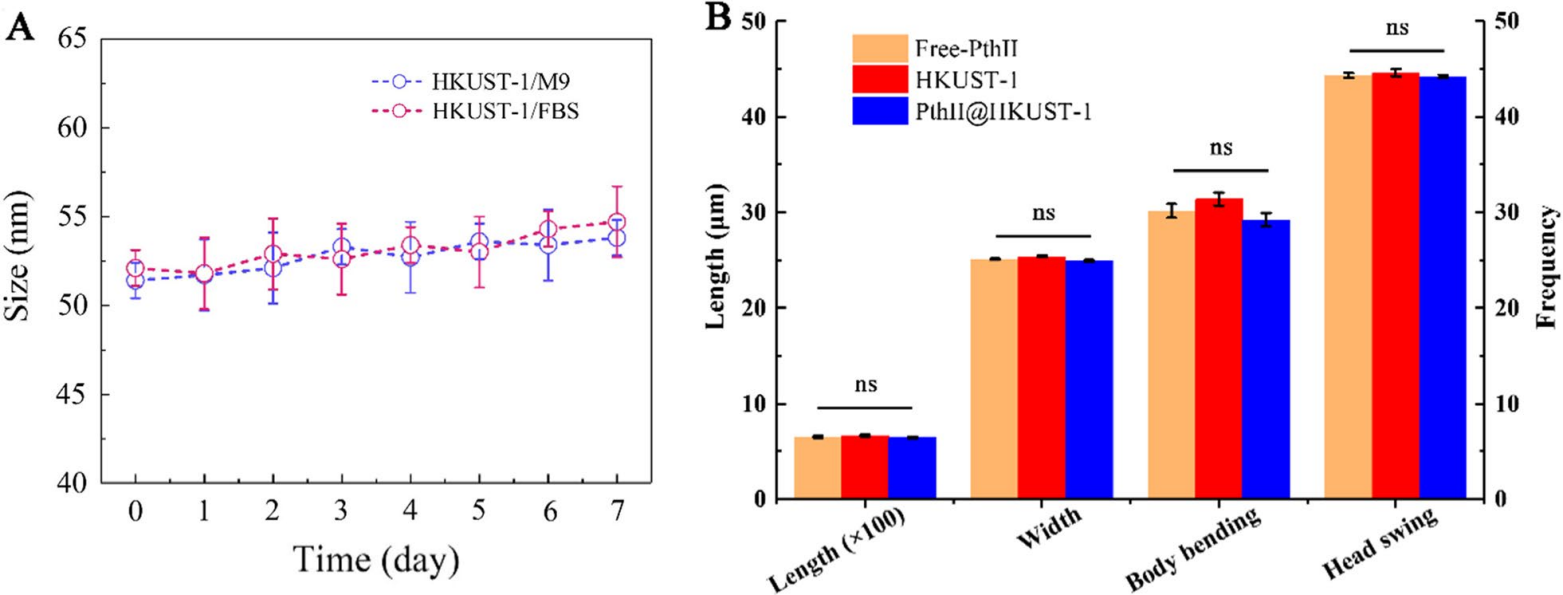
**A** Size stability of HKUST in M9 buffer and 10% FBS for 7 days; **B** the relevant parameters of *C. elegans* after Free-PthII, HKUST-1 and PthII@HKUST-1 treatment with concentration of 20 μg/mL, data in the figure are mean ± SD of ten replications (n = 10), ns indicate no significant differences (ANOVA, *p* > 0.05)

Benefiting from the advantages of *Caenorhabditis elegans* has short life cycle, complete genome, easy reproduction, etc., it was often used to assess the acute toxicity and lethal effects of heavy metals, organics and pesticide, and was widely used in environmental assessments such as soil and wastewater [34, 35]. The model organism *C. elegans* was as the object to initially assess the impact of carrier on the environment. It can be seen from Fig. 10A, the size of HKUST-1 has little change in M9 buffer and 10% FBS within 7 days, indicating that HKUST-1 have good stability in M9 buffer and 10% FBS and can be used for environmental assessment. The acute toxicity test result showed that the LC_50_ of PthII@HKUST-1 to *C. elegans* was 806.82 μg/mL, which implied very low in acute toxicity. The effect on the body length, width and mobility of *C. elegans* under the conventional application concentration of 20 μg/mL were explored. As shown in Fig. 10B, there was no change in the body shape of *C. elegans* after PthII@HKUST-1 treatment, and there was no significant difference in body length and width from the PthII@HKUST-1 treatment.

At the same time, the body bending frequency and head swing frequency of the HKUST-1 treatment group were slightly higher than that of the PthII@HKUST-1 treatment group, but the difference was not significant. The above results showed that PthII@HKUST-1 treatment has no effect on the body shape and activity ability of *C. elegans*, and it could be considered that PthII@HKUST-1 was an environmentally safe carrier material. There are reports on the application of HKUST-1 to the development of medical drugs [36], but the impact of HKUST-1 on the environment has not been clarified. There was no effect of HKUST-1 and PthII@HKUST-1on the important soil model microorganism *C. elegans* at regular concentrations, and was safe for soil microorganisms. It could be considered for the development of chemical pesticide carriers.

### Transport efficiency

**Fig. 11:**
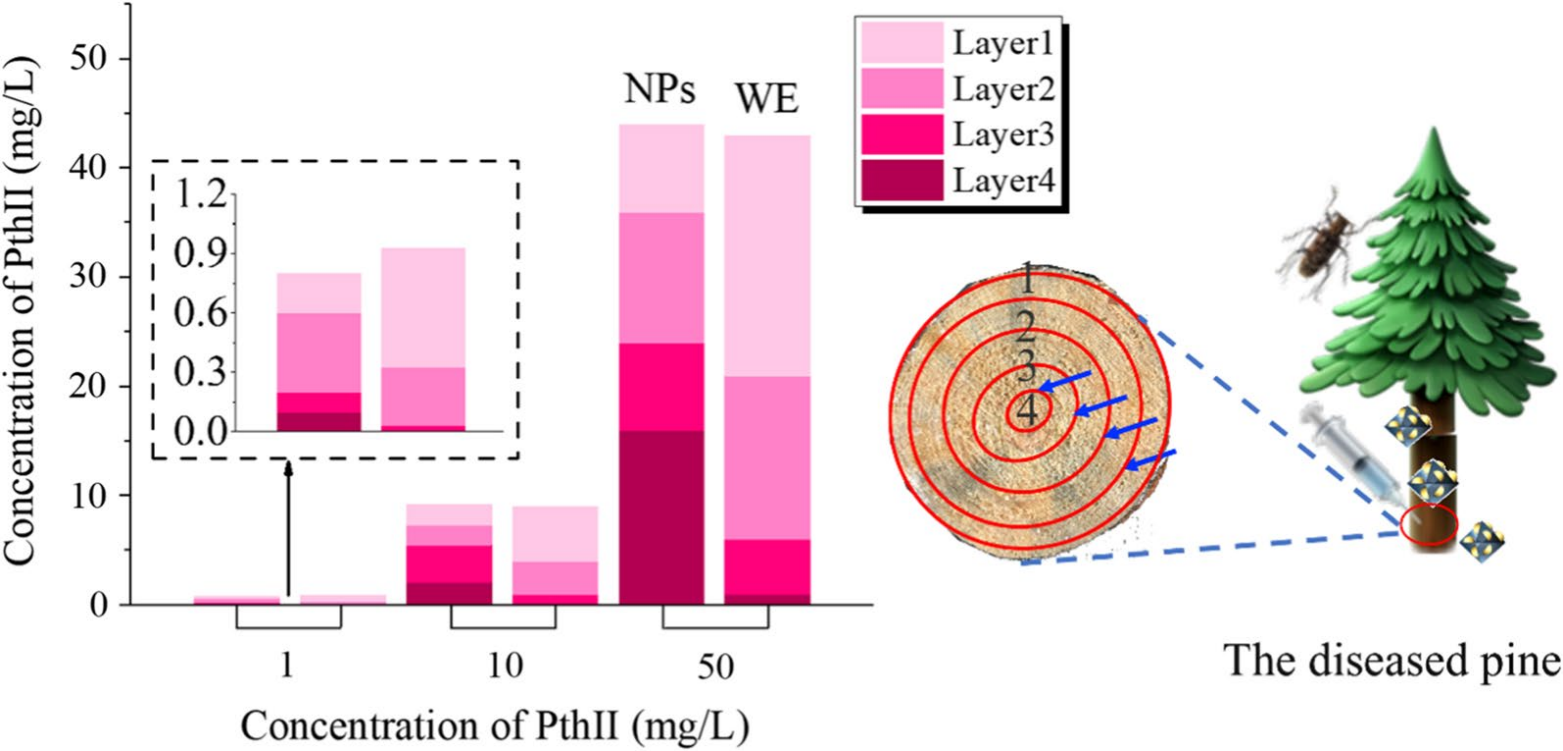
The content of PthII in the 1–4 layers of the pine trunk changes after injecting with PthII@HKUST-1, and PthII/WE as a control

**Fig. 12:**
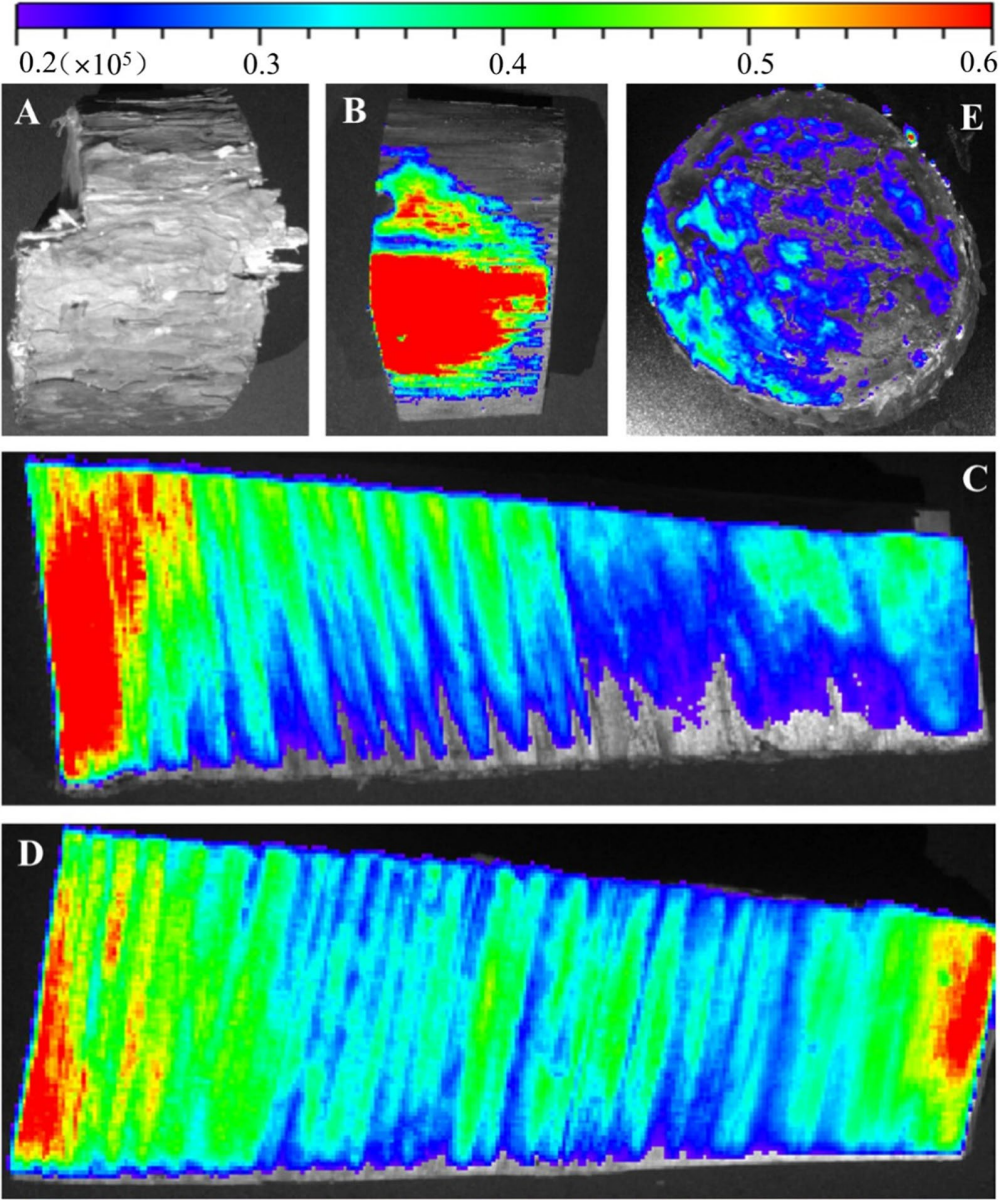
Tree trunks injected with water (**A**), fluorescence distribution of pine trunk after peeling off the bark (**B**), fluorescence intensity along the pith direction after 5 h (**C**), fluorescence intensity along the pith direction after 24 h (**D**), and fluorescence distribution of the cross-sectional view of the trunk after 24 h (**E**)

**Fig. 13:**
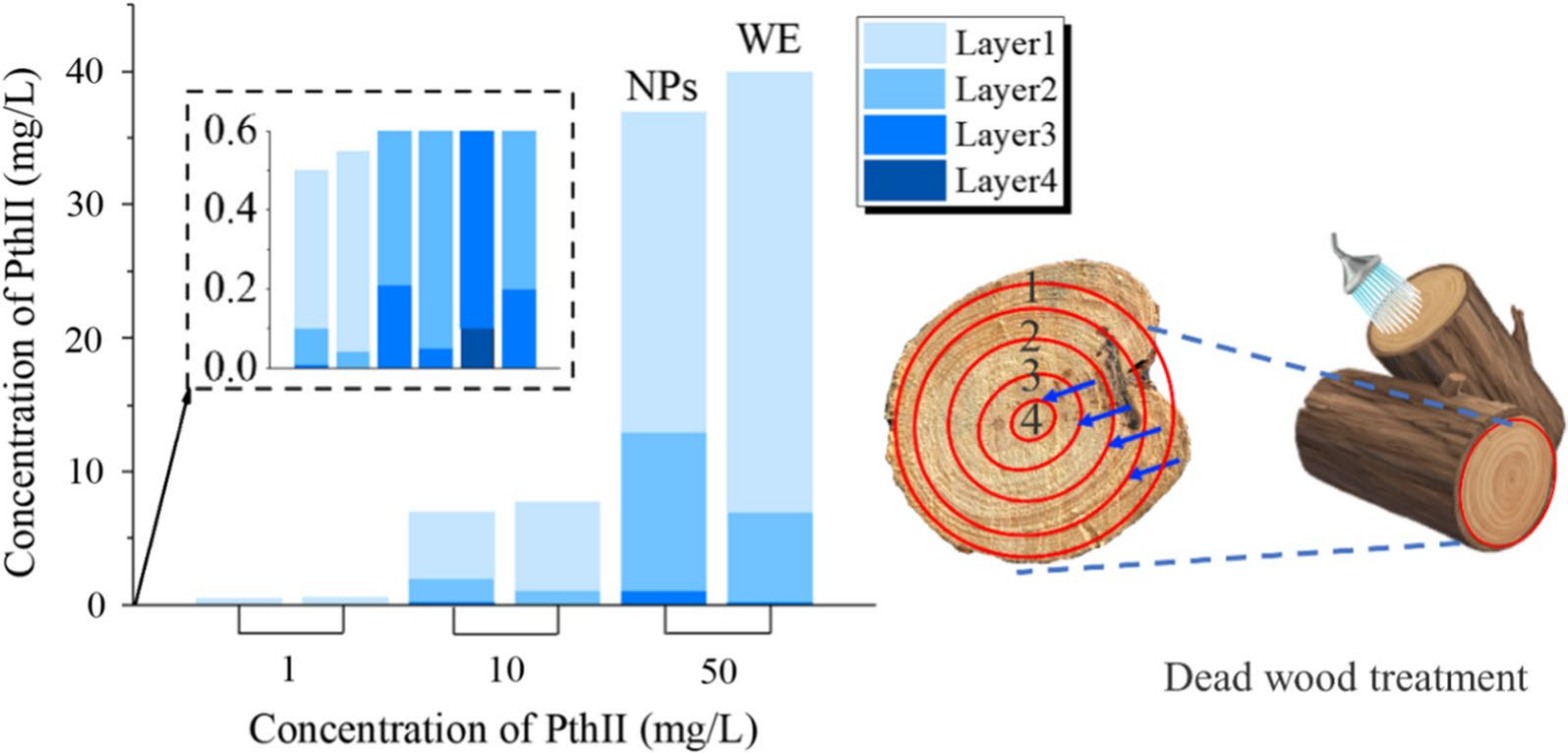
The content of PthII in the 1–4 layers of the dead wood after injecting with PthII@HKUST-1, and PthII/WE as a control

Because the outer epidermis of the tree is dead tissue, it is composed of keratinized cells, produced by cork cambium. Cork is the main component of the outer layer of the bark, which can insulate moisture and gas from passing through, protect the tree, and is a natural barrier for chemical control. Therefore, we deliver nano-biopesticide into the tree by injection. PthII@HKUST-1 was designed to control BX and its vector insect MA, and the transportation efficiency in the pine tree trunk would directly affect its application prospects. As shown in Fig. 11, At the same concentration, the transmission efficiency of PthII@HKUST-1 is significantly higher than that of PthII/WE. At the same concentration, the transmission efficiency of PthII@HKUST-1 was significantly higher than that of PthII/WE. The higher the concentration of PthII@HKUST-1, the higher the concentration of the biopesticide reaching the pith. PthII@HKUST-1 could be transmitted to the tree pith at a low concentration of 10 mg/L with the transport of water, while it is obviously difficult for PthII/WE to reach the tree pith. This phenomenon can be further verified from Fig. 12. We introduced FITC@HKUST-1 (equivalent mass of FITC and PthII, 20 mg/L, 100 mL) into the tree by punch injection, and achieved the delivery of nano-pesticide through water transport in the tree. Compared to tree trunks injected with water (Fig. 12A), the strong fluorescence distribution was observed by peeling off the bark (Fig. 12B), and fluorescence intensity decreases gradually along the pith direction (Fig. 12C). However, with the extension of the delivery time, we unexpectedly found that the fluorescence at the pith began to be enriched (Fig. 12D). What is the reason for this phenomenon? We speculated that the rate of water transport along the pith was different, the closer to the pith tree, the faster the water transport, and the greater of the biopesticide concentration difference between inside and outside, which lead to the continuous transport of FITC@HKUST-1 to the pith. This will also inhibit the irreversible damage caused by the migration of nematodes to the middle of the tree, indicating that PthII@HKUST-1 will effectively protect the main part of the tree from pests. In addition, the fluorescence distribution of the cross-sectional view of the trunk can observe the vertical and horizontal distribution of FITC@HKUST-1 (Fig. 12E). The treatment of dead wood was also very important during transportation. In this article, PthII@HKUST-1 was injected into the dead wood and sprayed PthII@HKUST-1 (adhesion of nanoparticles) on the bark surface to seal the dead wood. It can be seen from Fig. 13 that PthII@HKUST-1 could also be transmitted in dead wood. However, the transmission efficiency in dead wood at the same concentration was much lower than that of epidemic wood, and the same was true for PthII/WE transmission. This phenomenon can be understood as the basic stagnation of the water transport in the dead wood, the delivery of PthII@HKUST-1 was blocked, and efficient diffusion cannot be achieved in the dead wood, and the only water remaining in the dead wood was provided for the delivery of PthII@HKUST-1 effective support, which was also based on the good diffusion performance of HKUST-1 to promote the further transmission of PthII@HKUST-1.

## Conclusions

In summary, we put forward a proposal for utilizing PthII@HKUST-1 on prevention and control pine wood nematode and vector insect MA, including toxicity mechanism research, traceable biopesticide monitoring, and environment assessment for the first time. In order to solve the problems of poor water solubility, easy degradation and low control efficiency of PthII, the highly biocompatible material nanoHKUST-1 was used as a carrier for embedding PthII (biopesticide loading efficiency reached 85%) to prepare controlled release nano-pesticides. The particle size advantage of nanoHKUST-1 (50 nm) made it easily penetrate the body wall of MA larvae and transmit to tissue cells through contact and diffusion. In addition, PthII@HKUST-1 can effectively enhance the cytotoxicity and utilization of PthII, which will provide valuable research value for the application of typical plant-derived nerve agents in the prevention and control of forestry pests. Moreover, PthII@HKUST-1 could be transmitted to the epidemic wood and dead wood at a low concentration. We speculate that the multifunctional designed PthII@HKUST-1 will initiate new curiosity and enthusiasm in studying the uptake/inhibition mechanism of pine wood nematode and vector insects, and guide specific target genes. This will break through the limitations of existing research on pine wilt disease and related forestry pests.

## Data Availability

All data generated or analyzed during this are included in this published article.
